# Novel variant c.92T > G (p.Val31Gly) in the *PFN1* gene (ALS18) responsible for a specific phenotype in a large Bulgarian amyotrophic lateral sclerosis pedigree

**DOI:** 10.3389/fneur.2023.1094234

**Published:** 2023-02-09

**Authors:** Teodor Angelov, Teodora Chamova, Slavena Atemin, Tihomir Todorov, Slavko Ormandzhiev, Ivan Tourtourikov, Albena Todorova, David Devos, Ivailo Tournev

**Affiliations:** ^1^Department of Neurology, Faculty of Medicine, Medical University of Sofia, Sofia, Bulgaria; ^2^Genetic Medico-Diagnostic Laboratory “Genica”, Sofia, Bulgaria; ^3^Department of Medical Chemistry and Biochemistry, Medical University of Sofia, Sofia, Bulgaria; ^4^Department of Medical Pharmacology, Expert Center of Parkinson's Disease, LICEND COEN Center, NS-Park/FCRIN Network, Univ. Lille, Lille Neuroscience and Cognition, Team DVCD, INSERM UMRS_1172, CHU Lille, Lille, France; ^5^Department of Cognitive Science and Psychology, New Bulgarian University, Sofia, Bulgaria

**Keywords:** ALS, genetics, phenotype, pedigree, *PFN1*

## Abstract

**Objectives:**

Amyotrophic lateral sclerosis (ALS) is a neurodegenerative disorder characterized by progressive deterioration of motor function, disability, and death. Variants in the *PFN1* gene, encoding the Profilin-1 protein, are related to ALS18.

**Methods:**

We present a pedigree consisting of 3 generations and 4 affected individuals, 3 of which carry a novel heterozygous variant: c.92T > G (p.Val31Gly) in the *PFN1* gene. This variant was discovered through means of whole exome sequencing (WES) and targeted analysis of ALS-related genes.

**Results:**

The mean age of onset in our pedigree was 59.75 (±10.11 SD) years with a significant difference between the first two generations (females) and the third (male) of 22.33 (±3.4 SD) years. For this ALS form, we observed a longer disease progression of 4 (±1.87 SD) years (three of four affected are still alive). Clinical manifestations displayed predominant impairment of the lower motor neuron (LMN) in one limb, with gradual involvement of other limbs. A novel heterozygous missense variant c.92T > G, p. Val31Gly (NM_005022.4) in exon 1 in the *PFN1* gene was discovered through means of whole exome sequencing (WES). Segregation analysis in the family showed that the detected variant was inherited from the affected mother, and the affected aunt also turned out to be a variant carrier.

**Conclusions:**

ALS18 is a very rare form of the disease. We report here a relatively large pedigree with a novel variant, leading to late onset (after 50 years), initial involvement of the lower limbs and relatively slow progression.

## 1. Introduction

Amyotrophic lateral sclerosis (ALS) is a neurodegenerative disorder characterized by progressive deterioration of motor function, disability and death (1). The disease is genetically heterogeneous and can be caused by variants in certain genes (36 at the moment, according to latest data), encoding proteins involved in the development and metabolism of motoneurons (2). Clinical cases can be divided in two major groups—sporadic ALS (sALS) and familial ALS (fALS), of which 90–95 and 35–40% of cases respectively, are left without genetic verification.

The *PFN1* gene encodes the protein Profilin-1, pathogenic variants in which were found to cause ALS18 (3). Profilin-1 regulates the polymerization of actin, in response to extracellular signals, and thus is actively involved in cytoskeletal dynamics and axonal transport of motoneurons (4). As a result of the variations, monomeric globular (G)-actin cannot polymerize to filamentous (F)-actin (5). The reduced G/F-actin ratio, as well as reduced number of growth cones, are considered as a biochemical marker, when observing motoneurons at the microscopic level.

The first description of ALS18 was made by Wu et al. (3), who reported 22 patients with ALS, carrying 4 missense variants [c.211T > G (p.Cys71Gly); c.341T > C (p.Met114Thr); c.350A > G (p.Glu117Gly) and c.353G > T (p.Gly118Val)] in the *PFN1* gene. In this report the mean age of onset was 44.8 years, with initial weakness and atrophies in the limbs. In the following year, screening studies of the *PFN1* gene were performed and a new variant was demonstrated by Ingre et al. (6) [c.326C > T (p.Thr109Met)] in a German patient. A new variant was found by Chen et al. (7) [c.406C > T (p.Arg136Trp)] in a Chinese patient, and a single variant in the *PFN1* gene has been reported from the Middle East [c.58G > A (p.Ala20Thr)] by Smith et al. (8). Two studies also indicated that a variant in the *PFN1* gene [c.318_319dup (p.Asp107fs)] causes an early onset, polyostotic Paget-like disorder (9, 10).

## 2. Methods

**Figure 1:**
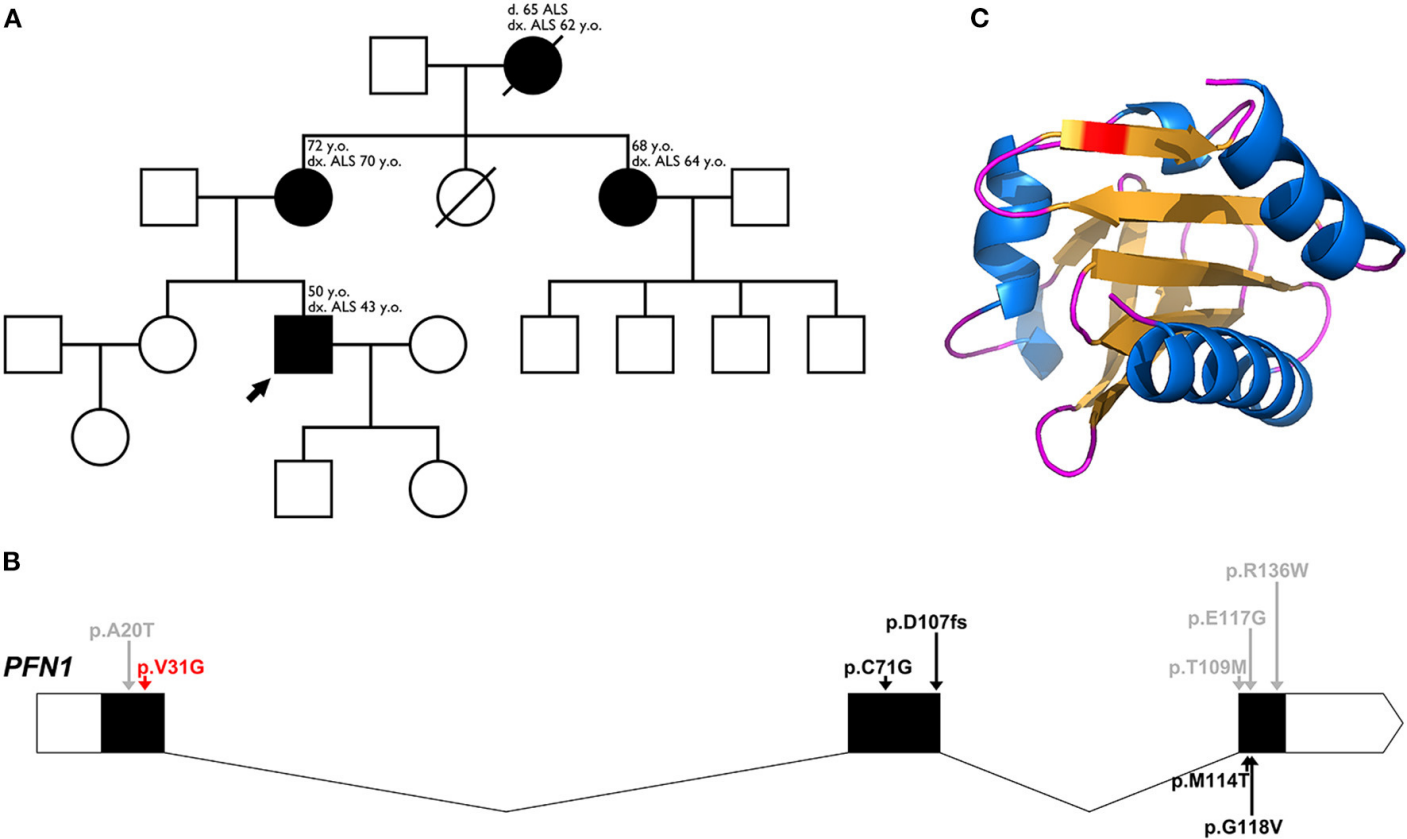
**(A)** Pedigree family tree for our family with 4 affected individuals. The proband of the family is marked with an arrow. For each affected individual, the age of onset and current age is indicated, as well as age of death for the deceased grandmother. **(B)** Schematic representation of the genomic structure of the *PFN1* gene, with intron-exon boundaries. 5′-3′ UTRs are indicated in white, coding regions are indicated in black and noncoding regions are indicated as thin arrow lines. Arrows indicate major variants, identified in ALS, as well as one variant associated with a Paget-like disorder (red arrow for our novel variant, black arrows for pathogenic/likely pathogenic variants, and gray arrows for variants of uncertain significance). **(C)** Three-dimensional structure of the altered sequence of human PFN1 protein, modeled with AlphaFold2.

We present a family, consisting of 3 generations and 4 affected individuals, with a clinical diagnosis ALS (Figure 1A).

All patients underwent neurological examination, electromyography (EMG), electroneurography (ENG), magnetic resonance imaging (MRI) of the brain and spine, evaluation by an otolaryngologist to assess bulbar dysfunction and neuropsychological assessment. Patients were assessed using the most common scale to determine the degree of UMN and LMN damage—ALS Functional Rating Scale-Revised (ALSFRS-R; Table 1).

**Table 1:** Epidemiological data and ALSFRS-R score for the affected patients.

				**Age at**	**Age at**			
		**Current**	**Age of**	**time of**	**time of**	**Disease**	**Age of**	**ALSFRS-R**
	**Sex**	**age**	**onset**	**clinical**	**genetic**	**duration**	**death**	**score**
				**diagnosis**	**diagnosis**			
III-2 (proband)	M	50 years	43 years	45 years	50 years	7+ years	–	36/48
II-1 (mother)	F	72 years	70 years	72 years	72 years	2+ years	–	43/48
II-3 (aunt)	F	68 years	64 years	68 years	68 years	4+ years	–	43/48
I-1 (grandm.)	F	–	62 years	63 years	–	3 years	65 years	N/A

Venous blood was taken for genetic testing (whole exome sequencing (WES) and targeted analysis of ALS-related genes).

Next generation sequencing—whole exome sequencing (NGS-WES, Illumina) was applied for evaluation of ALS related genes, associated with clinical symptoms of the target patient. Data interpretation was performed with the Software Gensearch NGS (Phenosystems). The detected variant in the *PFN1* gene was confirmed and studied for segregation in the family by Sanger sequencing.

Sanger sequencing of the targeted gene was performed with the Big Dye^®^ Terminator cycle sequencing kit v.3.1 (Applied Biosystems). Sequencing profiles were interpreted with the Sequencing Analysis v.5.1.1 software.

The study complies with the ethical guidelines of the institutions involved.

## 3. Results

A novel heterozygous variant: c.92T > G (p.Val31Gly), in the *PFN1* gene, was found in 3 of the affected individuals (one of the affected individuals passed away before the conducted genetic tests) (Figure 1B). The novel variant was found in the proband (onset of disease at the age of 43, currently 50 years old) and segregation analysis was conducted for the remaining affected individuals—his mother (onset of disease at the age of 70, currently 72 years old) and his aunt (onset of disease at the age of 64, currently 68 years old). The grandmother of the proband had an onset of disease at the age of 62, and died at the age of 65 before the genetic tests were conducted. All of the affected members are of Bulgarian ethnicity and no healthy family members were available for genetic testing. Occupational risk factors were not observed, as patients were not professionally engaged in heavy physical labor or in an environment with toxins and industrial hazards.

The epidemiological and clinical features of the affected patients are presented in Tables 1, 2.

**Table 2:** Impairment of the UMN and LMN by regions.

		**Neuronal impairment by regions:**						
			**LMN**	**LMN**	**LMN**			
	**Sex**	**LMN brain**	**cervical**	**thoracic**	**lumbar**	**Pyramidal**	**Impairment of LMN syndrome**	**Impairment of UMN**
		**stem**	**region**	**region**	**region signs**	**syndrome**		
**III-2** **(proband)**	M	–	+	–	+++	+	Lower flaccid paraplegia, equally pronounced in the proximal and distal muscle groups, impossible independent gait, muscle hypotrophy, more pronounced in the lower extremities (proximal and distal muscle groups) and left arm (mainly in the proximal muscle groups), weakened knee and Achilles reflexes bilaterally	Positive pathological Hoffmann and Tromner reflexes bilaterally, positive Babinski's sign on the left
**II-1** **(mother)**	F	+	–	–	++	–	Muscle weakness in lower extremities (left > right) for proximal and distal muscle groups, impossible independent gait, absent knee and Achilles reflexes in left leg; weakened pharyngeal reflex on the left, muscle fibrillation on the tongue	–
**II-3** **(aunt)**	F	–	–	–	++	+	Muscle weakness in the left lower limb, more pronounced in the proximal muscle groups, muscular hypotrophy in the left femoral muscles and slightly pronounced in the muscle groups thenar and m. interosseus dorsalis, bilaterally, impossible independent gait, missing knee and Achilles reflexes on the left	Positive pathological Marinescu-Radovici reflex bilaterally
**I-1** **(grandm.)**	F	–	–	–	+++	–	N/A	N/A

The mean age of onset in our family is 59.75 (±10.11 SD)−62 years for generation I, 67 years for generation II and 43 years for generation III, with a significant difference between the first two generations (females) and the third (male) of 22.33 (±3.4 SD) years. The mean disease duration is 4 (±1.87 SD) years, as of making of this article (three of four affected individuals are still alive). Neurological assessment was consistent, with LMN involvement predominantly in the lower limbs, and milder involvement in the upper limbs for the proband. His mother had bulbar involvement as well, consisting of weakened pharyngeal reflex on the left and muscle fibrillation on the tongue.

The ALSFRS-R scale for all affected individuals showed evidence of predominant LMN impairment at the lower limb region. The mother has also discrete involvement in the brainstem, which does not affect her daily functions. There was no significant reduction in daily functions due to impairment of the UMN. These data explain the relatively high points score on this scale (40.66/48), and if we take into account the lower sum of the proband (36/48), the mother's and aunt's functions are largely preserved.

A novel heterozygous missense variant: c.92T > G (p.Val31Gly) (NM_005022.4) in exon 1 of the *PFN1* gene was found by WES. Based on the standards and guidelines of ACMG/AMP for interpretation of sequence variants (11), we currently classified it as likely pathogenic (categories: PP1, PP3, PP4, and PM2). The variant is not found in gnomAD genomes and exomes. The CADD score for the variant is 32, with REVEL scoring at 0.895. In total, 11 *in silico* predictors classify the variant as pathogenic and 7 classify it as likely pathogenic. The variant was confirmed by Sanger sequencing, and segregation analysis in the family showed that the detected variant is inherited from the affected mother, and the affected aunt also turned out to be a variant carrier.

The variant is located within the critical Profilin-1 domain, between amino acids 2-106. Simulating the altered sequence, using AlphaFold2 (12), did not show any structural alterations (Figure 1C). While the actin interaction site of PFN1 lies within amino acids 60-130, there have been reports of likely pathogenic/pathogenic variants outside of this sequence. The valine residue at position 31 is highly conserved among several species, including yeast, rat and nematode. The neighboring positions 30 and 32 are highly conserved as well, and act as poly-proline binding sites.

## 4. Discussion

ALS18 is a very rare form of ALS. The pedigree reported by us carries a novel, and so far undescribed variant, c.92T > G (p.Val31Gly), which is characterized by specific clinical features, consistent with the ALS18 form. In the same exon 1 of the *PFN1* gene, apart from the detected in our study variant, two non-pathogenic (synonymous) variants have also been detected [c.93C > G (p.Val31Val) and c.93C > T (p.Val31Val), gnomAD browser].

Furthermore, ALS18 is distinguished by a specific phenotype: an age of onset after 50 years, an initial region of involvement in the lower extremities (and very rarely in the upper extremities) and long-term disease course without severe disability. These hallmarks should be carefully considered in newly diagnosed patients, as they largely resemble those of sporadic ALS cases, especially ones without symptoms, characterized by late onset and a long clinical course, or in cases, which are often interpreted as secondary ALS-syndrome (due to disc pathology or other causes). From this point of view, ALS18 may not be as rare, if a large number of cases have been misdiagnosed. An additional factor in their potential underestimation is the fact that increasingly genetic data is accumulating for variants in non-coding regions of the genome (e.g., introns, 5′ and 3′ untranslated regions etc.), defects in which can also lead to disease development (13–15).

ALS18 should be taken into account in cases with main key features, such as late age of onset (after 50 years) and/or initial region of involvement in the lower extremities (with or without discrete migration in other regions), and/or long-term course of the disease without severe disability significantly impairing daily functions.

We observed discrete differences in the epidemiological and clinical data for our described family, and for previously described patients with ALS18 (Table 3). The novel variant, described in this study [c.92T > G (p.Val31Gly)], allows to further strengthen the association of pathogenic variants in the *PFN1* gene (which have been reported to induce disturbed dynamics of microtubules and/or troubled coordination between actin and microtubule filaments in motoneurons) with development of ALS18 (16). Different variants in the *PFN1* gene lead to different flexibility and stability of microtubule entities, reported in a recent study using proteomic analysis in mammalian cells (17). According to this study, the variants c.341T > C (p.Met114Thr) and c.353G > T (p.Gly118Val) are indicated as partially destabilizing the Profilin-1 protein, while the variant c.211T > G (p.Cys71Gly) leads to severe destabilization at the microtubule level, due to its significant instability and increased accumulation of insoluble fractions.

**Table 3:** Epidemiological and clinical characteristics, with comparison of our novel variant and others described in scientific literature.

					**Difference**					
					**in age of**			**LMN**		
		**No of**		**Mean age**	**onset b/w**	**Disease**	**Initially**	**spinal**	**UMN**	**Brainstem**
**Variants**	**Origin**	**patients**	**Sex**	**of onset**	**F and M**	**duration**	**involved**	**segments**	**impairment**	**impairment**
					**patients**		**regions**	**impairment**		
c.92T > G (p.Val31Gly)	Bulgaria	4	3 F 1 M	59.75 years	Significant difference b/w F and M patients of 22.33 years	2, 4, and 7 years (only the grandmother is deceased, 3 years after onset)	Lower extremities	+	Discrete pyramidal symptoms in proband (positive pathological Hoffmann and Tromner reflexes bilaterally, positive Babinski's sign on the left)	Discrete bulbar symptoms in the mother and pseudobulbar symptoms in the aunt
c.211T > G (p.Cys71Gly); c.341T > C (p.Met114Thr); c.350A > G (p.Glu117Gly); c.353G > T (p.Gly118Val) Wu et al. (3)	Caucasian and Sephardic Jews	22	N/A	44.8 years	N/A	N/A	upper and lower extremities	+	–	–
c.326C > T (p.Thr109Met) Ingre et al. (6)	Germany	1	F	48 years	N/A	N/A	Lower extremities	+	Positive mandibular reflex and a positive Babinski's sign bilaterally	Bulbar syndrome and a need for non-invasive ventilation within 7 years after onset
c.406C > T (p.Arg136Trp) Chen et al. (7)	China	1	F	27 years	N/A	N/A	Extremities	+	–	–
c.58G > A (p.Ala20Thr) Smith et al. (8)	Middle East	1	F	63 years	N/A	At least 5 years	Extremities	+	Discrete signs of impairment	–

## 5. Conclusion

In this publication we describe a novel variant [c.92T > G (p.Val31Gly)] in the *PFN1* gene, responsible for development of ALS18. The family we report consists of 3 generations and 4 affected individuals, making it one of the largest with this form of ALS. The data reported by us help to broaden the clinical-genetic phenotype of ALS and to improve the differential-diagnostic notions, given its specific clinical phenotype.

## Data availability statement

The datasets presented in this article are not readily available because of ethical and privacy restrictions. Requests to access the datasets should be directed to the corresponding author.

## Ethics statement

The studies involving human participants were reviewed and approved by Ethics Committee at Medical University of Sofia. The patients/participants provided their written informed consent to participate in this study.

## Author contributions

TA, TC, TT, AT, DD, and ITourn: formal analysis (equal), investigation (equal), methodology (equal), writing-original draft (equal), writing-review and editing (equal), and conceptualization (equal). SA and SO: formal analysis (equal), investigation (equal), methodology (equal), writing-original draft (equal), and writing-review and editing (equal). ITourt: formal analysis and conceptualization. All authors contributed to the article and approved the submitted version.
